# Concurrent 12p trisomy and 4q34.2-q35.2 deletion detected by WES-CNV: Case report

**DOI:** 10.1097/MD.0000000000047434

**Published:** 2026-01-30

**Authors:** Xiaoyu Sun, Shuangzhu Lin, Wanqi Wang, Yangfan Qi, Kai Jiang

**Affiliations:** a College of Traditional Chinese Medicine, Changchun University of Chinese Medicine, Changchun, Jilin Province, China.

**Keywords:** 12p trisomy syndrome, 4q deletion syndrome, *FAT1*, global developmental delay

## Abstract

**Rationale:**

Genetic diagnosis of developmental delay remains challenging, particularly in cases with a negative karyotype. Trisomy 12p syndrome is attributed to dosage effects of critical genes on chromosome 12p, such as *KRAS* and *ETV6*. Specifically, germline duplication of *KRAS* – a key regulator of the mitogen-activated protein kinase signaling pathway *–* can disrupt neurodevelopment and skeletogenesis, while duplication of *ETV6* may alter its role in hematopoiesis and embryonic differentiation, contributing to the syndromic phenotype.

**Patient concerns:**

A 21-month-old boy presented with global developmental delay (GDD) as the primary concern.

**Diagnoses:**

Whole-exome sequencing detected a 35.8 Mb duplication on chromosome 12p concomitant with a 14.39 Mb deletion at 4q34.2-q35.2, leading to a diagnosis of concurrent 12p trisomy and 4q deletion syndromes.

**Interventions:**

The child exhibited severe craniofacial and upper limb deformities concomitant with GDD. A multidisciplinary symptomatic management plan was implemented, encompassing muscle strengthening exercises and structured language training. A custom dynamic forearm orthosis was prescribed to optimize functional limb positioning.

**Outcomes:**

At follow-up, the patient has developed independent sitting ability and improved upper limb functional mobility, though no significant change in humeral length was observed.

**Lessons:**

This study delineates a unique case of dual, presumed de novo copy number variants *–* 12p trisomy and 4q deletion *–* exhibiting synergistic pathogenesis. The resulting phenotypic superposition, characterized by extreme brachymelia and GDD, was more severe than what is typically associated with either anomaly alone. The origin of these copy number variants, while presumed de novo, could also plausibly result from an unbalanced translocation, a key limitation of this study. This case establishes a novel model for understanding syndromic developmental delay in the context of a negative karyotype and validates the superior diagnostic capability of whole-exome sequencing-based copy number variant analysis in identifying complex genomic disorders.

## 1. Introduction

Global developmental delay (GDD), defined as significant delay in at least 2 developmental domains, is a major pediatric health concern affecting 1% to 3% of children under 5 years of age and necessitates multidisciplinary early intervention.^[1]^ Tao et al^[2]^ identified chromosomal abnormalities in 30% of 99 children with developmental delay/intellectual disability. However, conventional karyotyping has limited resolution, failing to detect approximately 35% to 45% of pathogenic copy number variants (CNVs) larger than 5 Mb.^[3]^ Among the CNVs that evade karyotypic detection, both 12p trisomy syndrome (OMIM #600628) and 4q deletion syndrome (OMIM #613509) are rare disorders with distinctive syndromic features.

Trisomy 12p syndrome (incidence ~1:50,000) presents with distinctive craniofacial dysmorphism *–* such as hypertelorism and microphthalmia *–* alongside GDD, hypotonia, and seizure disorder.^[4]^ In contrast, 4q deletion syndrome exhibits a broad phenotypic spectrum, principally featuring skeletal anomalies (e.g., limb shortening and digital abnormalities), neurodevelopmental impairments, and behavioral disturbances.^[5]^ Copy number alteration profiling identified FAT1 (located at 4q35.2) as the most frequently altered gene by somatic deletions, occurring in 22.2% (4/18) of tumor samples.^[6]^

This report describes, to our knowledge, the first case of dual presumed de novo CNVs *–* a 12p trisomy and a 4q34.2-q35.2 deletion *–* identified by whole-exome sequencing (WES)-based copy number variant (CNV) analysis in a patient with a negative karyotype. The genomic origin of these CNVs may involve 2 distinct mechanisms: independent de novo events or, perhaps more likely, an unbalanced segregation from a parental balanced translocation, such as t (4;12); this uncertainty represents a study limitation. The synergistic interaction of these anomalies resulted in a severe composite phenotype, including extreme brachymelia and GDD, which was markedly more severe than could be attributed to either anomaly alone. This distinguishes the present case from previously reported instances involving single variants or familial deletions, underscoring the diagnostic utility of WES-CNV in resolving complex genomic disorders.

## 2. Case report

### 2.1. Chief complaint

A 21-month-old male infant presented for evaluation of GDD persistent since 6 months of age.

### 2.2. History of present illness

The patient, initially evaluated at 15 months of age, exhibited GDD. Key milestones were significantly delayed: head control was not achieved by 6 months, independent sitting by 12 months, or independent walking by 21 months. Purposeful vocalizations were absent by 18 months, alongside delayed social responsiveness. There was no reported history of seizures or feeding difficulties. Despite multiple prior evaluations at local hospitals and a diagnosis of “developmental delay” with symptomatic treatment, no clinical improvement was observed. He was subsequently referred to and admitted through our outpatient clinic for further investigation of “global developmental delay,” with persistent symptoms encompassing motor delay (nonambulatory), absent speech, and impaired social interaction.

### 2.3. Past medical history

The patient has no significant medical history, including infection or trauma. The mother’s obstetric history is G2P2 (gravida 2, para 2), with a full-term cesarean delivery and a birth weight of 2.8 kg. There was no neonatal asphyxia, and the patient was exclusively breastfed until 6 months of age.

### 2.4. Personal and family history

Both parents are healthy with no personal or family history of deformities, developmental abnormalities, or heritable conditions. The father exhibits normal upper limb proportions. The mother reported an unremarkable pregnancy, denying any significant medication use or known teratogenic exposures. No similar phenotypes have been reported in the family.

### 2.5. Physical examination

Height 78 cm, weight 9.5 kg, and Head circumference 46 cm. The patient was alert and responsive with good mental status. Dysmorphic facial features included wide-set eyes (inner canthal distance 3.5 cm), bilateral epicanthal folds, narrow palpebral fissures (longitudinal diameter 2.0 cm), a high-arched palate, and low-set, posteriorly rotated ears. The upper limbs were shortened (humerus 8 cm; radius 7 cm), with bilateral flexion of the little fingers and hypoplasia of the middle phalanges. Neurologically, the patient presented with generalized hypotonia and diminished tendon reflexes; no pathological reflexes were elicited.

### 2.6. Laboratory tests

Complete blood count, liver and kidney function, thyroid function, blood ammonia/lactate levels all normal.

### 2.7. Further diagnostic testing

Routine G-banding karyotype analysis revealed a normal 46, XY male karyotype. To further investigate the etiology, trio-based WES was performed on the Illumina NovaSeq 6000 platform, achieving an average coverage depth of 100×. CNV analysis identified 2 pathogenic CNVs: a ~35.8 Mb partial trisomy of chromosome 12p (chr12:1–3,58,00,000) and a ~14.39 Mb deletion at 4q34.2-q35.2 (chr4:17,65,56,055–190,948,359). Parental segregation analysis confirmed that both variants were of presumed de novo origin. However, the possibility of an inherited unbalanced translocation stemming from a parental balanced translocation, t (4;12), could not be definitively excluded due to the unavailability of parental samples for <confirmatory cytogenetic studies.

This case report describes a 21-month-old Chinese male presenting with a complex phenotype including GDD, severe craniofacial dysmorphism (including hypertelorism, epicanthal folds, and a high-arched palate), and profound skeletal abnormalities predominantly characterized by upper limb brachymelia. Initial G-banding karyotyping showed a normal 46, XY result. Subsequent family-based WES-CNV analysis identified dual pathogenic CNVs: a ~35.8 Mb duplication indicative of trisomy 12p and a ~14.39 Mb heterozygous deletion at 4q34.2-q35.2.

Parental segregation analysis confirmed that both CNVs were apparently de novo in origin. However, the possibility that they resulted from an inherited unbalanced translocation stemming from a parental balanced translocation, such as t (4;12), could not be definitively excluded due to the unavailability of parental samples for karyotyping. The WES-CNV findings were thus diagnostic of concurrent 12p trisomy and 4q34.2-q35.2 deletion syndromes.

**Figure 1.**
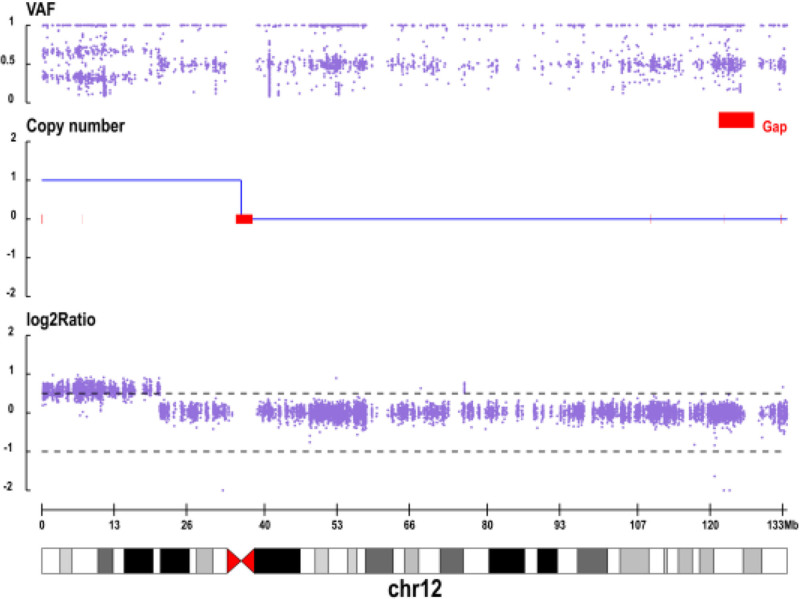
Proband (male, patient) in the copy number distribution map of chromosome. VAF = variant allele frequency.

**Figure 2.**
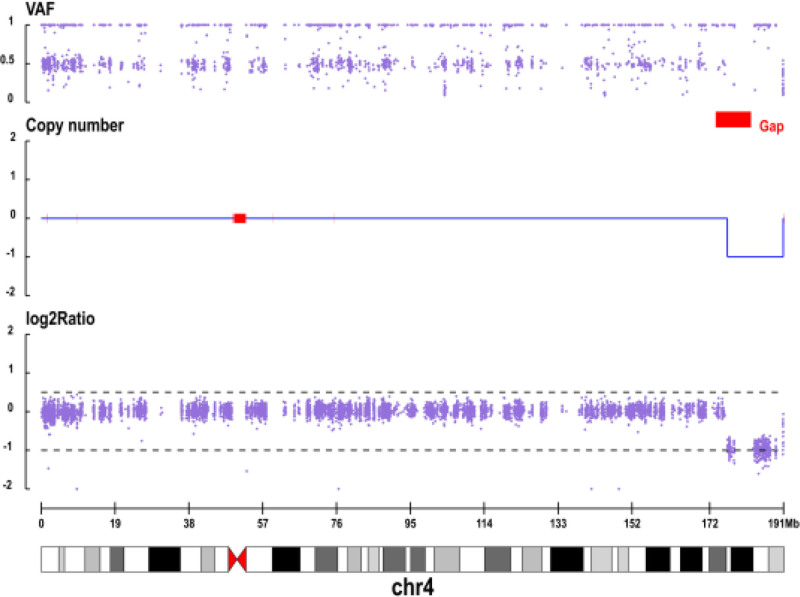
Proband (male, patient) in the copy number distribution map of chromosome 4.

Figure 1 shows a conspicuous increase in copy number across the short arm of chromosome 12, corresponding to a ~35.8 Mb duplication consistent with trisomy 12p. This finding correlates with the patient’s clinical features of GDD and craniofacial dysmorphism, which are characteristic of this syndrome. Similarly, Figure 2 reveals a distinct heterozygous deletion within the 4q34.2-q35.2 region, spanning approximately 14.39 Mb. This result aligns with the observed skeletal anomalies and neurodevelopmental issues associated with 4q deletion syndrome. The CNV profiles clearly delineate the genomic location and magnitude of these pathogenic variants, thereby providing crucial molecular evidence for a concurrent diagnosis of both disorders.

## 3. Results

WES-CNV analysis detected 2 pathogenic CNVs: a ~35.8 Mb partial trisomy of chromosome 12p and a ~14.39 Mb deletion at 4q34.2-q35.2. The proband exhibited extreme brachymelia that was markedly more severe than typically observed in isolated 4q deletion syndrome,^[5]^ providing phenotypic evidence of a synergistic interaction between these genomic events.

## 4. Discussion

This study reports a case of dual CNVs *–* a partial trisomy 12p and a 4q34.2-q35.2 deletion. Although parental segregation analysis suggested a de novo origin, the terminal locations of these anomalies raise the possibility of an unbalanced segregation from a parental balanced translocation, t (4;12). This uncertainty regarding the precise genomic mechanism represents a limitation of our study. Nevertheless, the proband exhibited severe skeletal and multisystem abnormalities consistent with synergistic pathogenesis.

We propose a synergistic pathogenesis model in which the dosage effect of trisomy 12p (e.g., *KRAS* overexpression) exacerbates the phenotypic consequences of haploinsufficiency due to the 4q deletion (involving genes such as *FAT1*). This interaction underlies the severe skeletal deformities observed, which exceed the typical phenotypic spectrum of either isolated anomaly.

The uniqueness of this case stems from the concomitant presence of 2 CNVs: a 12p trisomy and a 4q deletion. The patient’s clinical manifestations, particularly the extreme brachymelia, were markedly more severe than those typically associated with either anomaly alone, supporting a model of synergistic pathogenesis.

The differential diagnosis between the present case and Pallister–Killian syndrome (PKS), isolated 12p trisomy syndrome, and isolated 4q deletion syndrome was crucial, as shown in Table 1. PKS is typically characterized by a constellation of features including pigmentary skin anomalies, sparse scalp hair, distinctive craniofacial dysmorphism, and limb defects.^[7]^ Its underlying genetic etiology is mosaic tetrasomy 12p, often due to a supernumerary isochromosome i (12p). The present case lacked characteristic features of PKS, such as skin pigmentary anomalies or sparse hair. Crucially, genetic analysis identified a non-mosaic partial trisomy 12p, rather than a mosaic tetrasomy, thereby ruling out PKS.

**Table 1:** Phenotypic comparison between the present case and isolated anomalies or Pallister–Killian syndrome.

Feature	Present case (12p trisomy + 4q deletion)	Isolated 12p trisomy syndrome	Isolated 4q deletion syndrome	Pallister–Killian syndrome (PKS)
Genetic mechanism	Dual CNVs: partial 12p trisomy (~35.8 Mb) and 4q34.2-q35.2 deletion (~14.39 Mb); presumed de novo or potentially resulting from an unbalanced segregation of a parental balanced translocation, t (4;12)	Complete or partial trisomy 12p, usually de novo	Terminal or interstitial 4q deletion, can be de novo or inherited	**Mosaic tetrasomy 12p**, due to a supernumerary isochromosome 12p [i (12p)]; cytogenetic analysis often requires skin fibroblast culture rather than lymphocyte analysis due to tissue-specific mosaicism
Characteristic craniofacial features	Hypertelorism, epicanthal folds, high-arched palate, low-set and posteriorly rotated ears	Prominent forehead, hypertelorism, microphthalmia, broad nasal bridge, downturned corners of the mouth	Scalp defects (cutis aplasia), eyelid coloboma, cleft lip/palate, micrognathia	High, broad forehead; bitemporal depression; sparse eyebrows and eyelashes; **characteristic cutaneous pigmentary abnormalities (e.g., linear or patchy hyper-/hypopigmentation**)
Skeletal/limb anomalies	**Extreme upper limb brachymelia** (humerus 8 cm, radius 7 cm), flexed little fingers, shortened middle phalanges of the fifth fingers	Relatively mild; may include brachydactyly, polydactyly/syndactyly	Limb shortening (especially upper limbs), digital anomalies (e.g., syndactyly), hypoplastic nails	Limb asymmetry, brachymelia, polydactyly/syndactyly, joint contractures
Neurodevelopment	**Global developmental delay (GDD**), severe motor and language delay	Moderate to severe developmental delay/ intellectual disability (DD/ID), hypotonia, high risk of seizures	Mild to severe intellectual disability, developmental delay, behavioral issues (e.g., autism spectrum traits)	Moderate to severe developmental delay/ intellectual disability, seizures, hypotonia or hypertonia
Skin/appendages	No significant abnormalities reported	Nonspecific	Possible localized skin aplasia (especially on the scalp)	**Key differentiating feature: Congenital localized skin aplasia (especially of the scalp) is rare in PKS, whereas pigmentary abnormalities and sparse hair are more characteristic**
Diagnostic highlights/special points	**WES-CNV detected dual CNVs against a normal karyotype background**; phenotypic superposition with severity exceeding isolated anomalies	Large duplications may be detected by conventional karyotyping; phenotype is relatively typical	FISH or chromosomal microarray (CMA) can define the deletion extent; high phenotypic heterogeneity	**Highly dependent on detection method:** Conventional lymphocyte karyotyping may yield normal results; detection of the mosaic i (12p) often requires skin fibroblast culture or FISH/molecular genetic analysis on affected tissues.
Key differentiator for this case	Manifests a severe superposition of both syndromes, but **lacks the characteristic cutaneous pigmentary anomalies and sparse hair seen in PKS**, and exhibits a uniform partial trisomy rather than mosaicism	Not associated with extreme brachymelia	Not associated with the typical craniofacial features of 12p trisomy	This case lacks key PKS phenotypes (pigmentary anomalies, sparse hair), and the genetic mechanism (uniform trisomy vs mosaic tetrasomy) differs fundamentally

CNV = copy number variant, PKS = Pallister–Killian syndrome, WES = whole-exome sequencing.

Studies have delineated *FAT1*’s role in myogenesis and its genomic significance. Smith et al^[8]^ reported that *FAT1* expression begins during limb bud formation, where it may regulate myocyte polarity by suppressing Wnt signaling, thereby facilitating the coordination of myotendinous junction development. Consistent with this, Caruso et al^[9]^ observed in a mouse model that *FAT1* deficiency impairs myoblast migratory polarity, resulting in morphological defects in shoulder and facial muscles, including reduced rhomboid muscle volume, confirming *FAT1*’s critical role in muscle morphogenesis. From a genomic standpoint, copy number analysis by Qu et al^[6]^ mapped *FAT1* to chromosome 4q35.2, identifying somatic deletions in 4 of 18 tumor specimens (22.2%), which establishes it as one of the most frequently altered genes in this region.

A recent study in human embryonic stem cells has identified a novel role for *KRAS* in osteogenesis, where it suppresses matrix metalloproteinase-9 (MMP9) activity to facilitate extracellular matrix accumulation and mineralization.^[10]^ Furthermore, *KRAS* suppresses myofibrillar protein synthesis and accelerates proteolysis through a tripartite network involving metabolic reprogramming, inflammatory cascades, and immune remodeling, ultimately driving muscle atrophy.^[11]^ Separately, Al Zeyadi et al^[12]^ used functional genomics to establish a significant positive correlation between a chromosomal segmental gain at 12p12.1-p11.22 and *KRAS* gene amplification. They also identified that a 4q34.2-q35.1 deletion modulates *KRAS* pathway activity, thereby contributing to oncogenesis.

The diagnostic utility of WES-CNV analysis in this case highlights its value in resolving complex genetic disorders. This approach integrates the high resolution of WES with robust CNV analysis, allowing for the concurrent detection of point mutations and large CNVs. It proved particularly effective where conventional karyotyping yielded a normal result.^[13]^ In contrast to standard karyotyping, WES-CNV reliably identifies pathogenic CNVs larger than 5 Mb, addressing a significant proportion (~35–45%) of previously undiagnosed cases.^[3]^ Notably, in this karyotype-negative patient, WES-CNV successfully identified dual pathogenic CNVs, underscoring its potential as a first-line diagnostic tool for children with atypical developmental delay.

The pathogenesis of concurrent 12p trisomy and 4q deletion may involve a synergistic interaction between *KRAS* overexpression and *FAT1* haploinsufficiency. *KRAS* activation promotes proliferation and differentiation via mitogen-activated protein kinase signaling, with its excess dosage exacerbating skeletal defects. Concurrently, *FAT1* haploinsufficiency disrupts cell polarity and myogenesis.^[14,15]^ The combined effect of these alterations likely leads to a critical dysregulation of shared developmental pathways, thereby amplifying the overall phenotypic severity.

Functional validation in cellular or animal models is required to elucidate the *KRAS–FAT1* interaction and its combined impact on neurodevelopment and skeletogenesis. Such insights would advance the understanding of complex genetic disorders and inform the development of targeted therapeutic strategies.

WES-CNV analysis effectively resolves diagnostic challenges in karyotype-negative cases by enabling the detection of multiple CNVs, thereby demonstrating its utility as a valuable supplemental test for children with developmental delay or malformations following inconclusive karyotyping.

This study has several limitations. The precise breakpoints of the CNVs were not experimentally validated. Furthermore, without parental genetic studies, the definitive origin (de novo vs inherited) of the CNVs could not be established. Finally, the proposed model of synergistic pathogenesis, while plausible, remains speculative in the absence of functional validation.

For children with limb malformations and a normal karyotype, the specific combination of hypertelorism and upper limb brachymelia should prompt evaluation for concurrent 12p trisomy and 4q deletion. Additionally, families with a 4q deletion should be advised of the risk for progressive hypotonia, necessitating regular neuromuscular surveillance.

## 5. Conclusion

This study reports a rare case of concomitant CNVs *–* partial trisomy 12p and a 4q34.2-q35.2 deletion *–* broadening the known phenotypic spectrum of trisomy 12p syndrome. The synergistic interaction of these CNVs likely accounts for the extreme upper limb brachymelia *–* more severe than typically observed in isolated deletions *–* potentially through a novel pathogenic mechanism involving dysregulated crosstalk between the *KRAS*-mitogen-activated protein kinase and *FAT1* pathways. This case highlights the superior diagnostic resolution of WES-CNV for detecting large-scale CNVs and provides a rationale for its use in karyotype-negative patients with characteristic phenotypes. We recommend multiplex CNV screening in children presenting with severe limb malformations and developmental delay.

## Acknowledgments

We thank the patient and his family members for their contributions to this study.

## Author contributions

**Conceptualization:** Kai Jiang.

**Data curation:** Xiaoyu Sun, Wanqi Wang, Yangfan Qi.

**Formal analysis:** Xiaoyu Sun.

**Writing – original draft:** Xiaoyu Sun, Shuangzhu Lin.

**Writing – review & editing:** Shuangzhu Lin.
